# Cyclical Patterns Affect Microbial Dynamics in the Water Basin of a Nuclear Research Reactor

**DOI:** 10.3389/fmicb.2021.744115

**Published:** 2021-10-15

**Authors:** Valérie Van Eesbeeck, Ruben Props, Mohamed Mysara, Pauline C. M. Petit, Corinne Rivasseau, Jean Armengaud, Pieter Monsieurs, Jacques Mahillon, Natalie Leys

**Affiliations:** ^1^Microbiology Unit, Environment, Health and Safety Department, Belgian Nuclear Research Centre (SCK CEN), Mol, Belgium; ^2^Food and Environmental Microbiology Laboratory, Earth and Life Institute, Catholic University of Louvain, Louvain-la-Neuve, Belgium; ^3^Center for Microbial Ecology and Technology, Ghent University, Ghent, Belgium; ^4^Institute for Integrative Biology of the Cell (I2BC), CEA, CNRS, Université Paris-Saclay, Paris, France; ^5^Technological Innovations for Detection and Diagnosis Laboratory, CEA, Bagnols-sur-Cèze, France; ^6^Protozoology Research Group, Department of Biomedical Sciences, Institute of Tropical Medicine (ITG), Antwerp, Belgium

**Keywords:** nuclear reactor, ultrapure water, ionizing radiation, 16S rRNA amplicon sequencing, aquatic microbiome, extreme environment

## Abstract

The BR2 nuclear research reactor in Mol, Belgium, runs in successive phases of operation (cycles) and shutdown, whereby a water basin surrounding the reactor vessel undergoes periodic changes in physico-chemical parameters such as flow rate, temperature, and radiation. The aim of this study was to explore the microbial community in this unique environment and to investigate its long-term dynamics using a 16S rRNA amplicon sequencing approach. Results from two sampling campaigns spanning several months showed a clear shift in community profiles: cycles were mostly dominated by two Operational Taxonomic Units (OTUs) assigned to unclassified *Gammaproteobacterium* and *Pelomonas*, whereas shutdowns were dominated by an OTU assigned to *Methylobacterium*. Although 1 year apart, both campaigns showed similar results, indicating that the system remained stable over this 2-year period. The community shifts were linked with changes in physico-chemical parameters by Non-metric Multidimensional Scaling (NMDS) and correlation analyses. In addition, radiation was hypothesized to cause a decrease in cell number, whereas temperature had the opposite effect. Chemoautotrophic use of H_2_ and dead cell recycling are proposed to be used as a strategies for nutrient retrieval in this extremely oligotrophic environment.

## Introduction

Waters of nuclear facilities are constantly filtered and deionized to remove dissolved radionuclides, as well as impurities and ions that could become activated. They are also exposed to varying levels of ionizing radiation depending on their proximity with radioactive sources (up to 40 kGy h^–1^ as estimated in the primary circuit of the BR2 reactor during operation). These ultrapure waters thus represent a rather inhospitable environment for microorganisms. Yet, bacteria, fungi, and microalgae have previously been detected in cooling waters of such facilities, namely in Spent Nuclear Fuel Pools (SNFPs), where spent nuclear fuel is stored in racks under water in order to cool down before being disposed (Santo Domingo et al., 1998; Chicote et al., 2005; Masurat et al., 2005; Rivasseau et al., 2016). Microbes can be present in the form of planktonic populations or biofilms, which can adhere to metal surfaces in SNFPs, potentially leading to microbiologically influenced corrosion (MIC; Zhang et al., 1999; Giacobone et al., 2011; Smart et al., 2014). Although this phenomenon has been investigated, together with the radionuclide bioaccumulation potential of some bacterial strains (Jolley, 2002; Tisakova et al., 2013), these studies mainly focused on cultivation-based approaches at a single point in time to identify and characterize the detected microorganisms. However, only a small percentage of environmental bacteria can be cultivated under laboratory conditions (Whitman et al., 1998). This fundamentally limits the potential to investigate the dynamics of the community as a whole. Conversely, with the advent of the next-generation sequencing (NGS) platform and –omics techniques, it has become possible to analyze the DNA from all the members of a microbial population, hereby gaining insights into the entire community. As such, those techniques are starting to get implemented in the study of microbial communities within SNFPs and other watery environments in nuclear reactors (Bagwell et al., 2018; MeGraw et al., 2018; Foster et al., 2020; Petit et al., 2020; Ruiz-Lopez et al., 2020). Nevertheless, knowledge on the evolution of these communities over time is currently lacking.

Instead of the previously described SNFP microbiota (Santo Domingo et al., 1998; Zhang et al., 1999; Chicote et al., 2005; Masurat et al., 2005; Giacobone et al., 2011; Tisakova et al., 2013; Smart et al., 2014; Rivasseau et al., 2016; Bagwell et al., 2018; MeGraw et al., 2018; Foster et al., 2020; Ruiz-Lopez et al., 2020), this work focuses on a similar but more extreme (higher radioactivity) environment consisting of a water basin directly surrounding the vessel of a nuclear reactor. By using a 16S rRNA amplicon sequencing approach, it was possible to identify bacterial taxa that went undetected before. Only a single study investigated a similar environment using an equivalent approach, namely the cooling pool of a nuclear reactor in France (Petit et al., 2020), highlighting the novelty of this research. Moreover, instead of sampling a single time point, our sampling campaign covering several months included a time series spanning both active and inactive periods of reactor operation, allowing us to investigate the dynamics of the microbial population over time. As the reactor is running in different phases, where an active period (cycle) is interspersed with periods of shutdown for reactor maintenance, these data shed a new light on the microbial dynamics within this unique environment. Since the transition between cycle and shutdown phases is accompanied by a shift in physical parameters such as temperature and radiation, this sudden change in external conditions was hypothesized to influence the community composition and cell density.

## Materials and Methods

### Sampling Site and Sample Collection

The study was conducted at the BR2 nuclear reactor at the Belgian Nuclear Research Centre (SCK CEN) in Mol, Belgium. The sampling environment consisted of an open basin surrounding the reactor vessel (Supplementary Figure 1). The reactor successively goes through cycles of operation (e.g., production of radioisotopes for medical and industrial use) and shutdown of approximately 30 days each, which are associated with changes in physico-chemical parameters of the water such as flow rate, temperature, and radioactivity. For this study, two sampling campaigns were performed: in the first one, the basin water was studied for three consecutive cycles interspersed with shutdown periods over a period of 8 months (from September 06, 2016 to April 28, 2017). The second one was conducted 1 year later (from April 16, 2018 to June 11, 2018) where it was only monitored for one cycle. In this case, the cell density was also assessed for each sample.

The study of the reactor basin environment represented a challenge in terms of its low bacterial load combined with the presence of radioactivity. For sample collection, a filtration system was therefore designed to minimize the risk of radioactivity exposure and contamination during sampling in accordance with the ALARA principle (As Low As Reasonably Achievable) and implemented in a sampling glove box, routinely used to take samples from the basin and other watery environments (e.g., the primary and secondary cooling circuits). This also ensured that enough cell material could be collected for downstream analyses. Of note, filtration was chosen as a preferred method over centrifugation, as this resulted in a more effective and safer sample collection.

The system was composed of a stainless steel filter holder containing a 0,2 μm pore sized polyethersulfone (Supor^®^) filter membrane (Pall Corporation, Port Washington, NY, United States), connected to the corresponding tap on one side and a flow meter on the other side in order to measure the filtered volume (Supplementary Figure 2). Ten liters of water were filtered per sample, after which the filter membrane was retrieved from the system and stored at −20°C in a controlled area (for a period of 1–9 months) before DNA extraction to allow for radioactive decay.

### DNA Extraction and 16S rRNA Amplicon Sequencing

DNA was extracted from the filter membranes following a previously described protocol (Vilchez-Vargas et al., 2013) and subsequently purified using Amicon^®^ 30 kDa filter cartridges (Merck, Darmstadt, Germany). DNA concentrations were measured using a Quantus^TM^ fluorometer (Promega, Madison, WI, United States). For the 16S rRNA amplicon sequencing, the hypervariable V3-V4 region was chosen to specifically target bacteria (as opposed to fungi and/or Archaea) and the resulting amplicons were sequenced in two runs using V3 chemistry (2 × 300 bp) with the Illumina MiSeq platform (sequencing performed by Eurofins Genomics, Ebersberg, Germany). Positive (*Cupriavidus metallidurans* CH34 cultures filtered over the same filter membranes as previously mentioned) and negative controls (blank filter membranes and demineralized water) were also added as part of the experiment. They underwent the same DNA extraction procedure as the other samples.

### Sequencing Data Analysis

The data generated by the sequencing platform were demultiplexed and the datasets consisted of two separate fastq files (forward and reversed). The sequences were subsequently analyzed through the OCToPUS pipeline as described in Mysara et al. (2017): In short, reads went successively through pre-assembly denoising (Bankevich et al., 2012), contig assembly, quality filtering using mothur (Schloss et al., 2009), denoising using IPED (Mysara et al., 2016), chimera removal using CATCh (Mysara et al., 2015) and finally Operational Taxonomic Unit (OTU) clustering with 97% cut-off using UPARSE (Edgar, 2013). All resulting OTUs were taken into account for further analyses without applying a minimum abundance threshold.

The OTUs were then taxonomically assigned using the mothur *classify.seqs* command with the RDP dataset (version 16) as reference. In order to properly compare the samples from both campaigns combined, they were rarefied to the same read count as the smallest sample (11,683 reads), using the mothur *sub.sample* command. Although a large number of reads is typically removed during this process, this does not significantly affect the subsequent alpha and beta diversity analyses. Rarefaction curves were obtained by using the mothur *rarefaction.single* command and subsequently plotting the data in the R software. Sample metadata are included in Supplementary Table 1.

Alpha (Inversed Simpson index) and beta [Theta-YC distance calculation followed by non-metric multi-dimensional scaling (NMDS)] diversity analyses were also performed in mothur using the *summary.single, dist.shared*, and *nmds* commands. In order to correlate the OTUs and metadata with the NMDS dimensions, a Spearman rank correlation analysis was performed using the mothur *corr.axes* command. OTUs and metadata that were significantly correlated (*r* > | 0.6|, *p* < 0.05) with the represented NMDS axes are displayed on the NMDS plots. It must be noted that the structure of our data did not allow for the use of mixed models.

### Cell Counting

During the second sampling campaign, a cell counting method was used to study the population dynamics of the basin water across the two reactor operation conditions (cycle vs. shutdown). The method consisted of heterotrophic plate count, where 100 μl of basin water was directly spread onto R2A agar (Reasoner and Geldreich, 1985) plates in triplicates. The plates were incubated for 2 weeks at room temperature on a laboratory bench before colony counting. Flow cytometry was also considered as a cell counting method, but proved to be unreliable as the cell numbers during cycles dropped significantly, thereby making it impossible to distinguish between bacterial cells and instrument background. This meant that relative OTU abundances could not be corrected for absolute cell numbers.

### Physical Characterization of the Basin Environment

The physical parameters of the BR2 waters are constantly monitored using on-line measuring instruments. Relevant data for the bacterial community (conductivity, temperature, radiation, and flow rate) were retrieved from an in-house software (BIDASSE) database and organized according to the sampling time points.

### Chemical Analysis

Water samples from both cycles and shutdown periods from campaign 1 were collected to perform a chemical analysis of ecologically relevant compounds such as nitrate, phosphate, and carbon. The ions (nitrite, nitrate, phosphate, and acetate) were quantified through ion chromatography using a 930 Compact IC Flex (Metrohm, Herisau, Switzerland) with a Metrosep A Supp 16-250/4.0 column and a Metrosep A Supp 16 guard/4.0 column, using an eluent solution of 7.5 mM Na_2_CO_3_ and 0.75 mM NaOH. TIC (total inorganic carbon) and TOC (total organic carbon) were measured using a FormacsHT-I TOC analyzer (Skalar Analytical, Breda, Netherlands). O_2_, H_2_, and ROS were not measured in our system.

### Temperature Experiment

To assess the influence of temperature, another critical parameter, on the community, an experiment was designed to test its effect on cell number. Briefly, basin water was collected from the BR2 reactor during a shutdown period in an autoclaved glass container and incubated at 35°C (approximately the same temperature as observed during a cycle) for a period of 24 h. As a control, the same volume of water was kept at room temperature (21 ± 2°C) next to the incubator. After 24 h, a fresh sample was collected from the BR2 reactor (also at room temperature or 21 ± 2°C) in order to be compared with the control. Cell numbers for all three conditions were measured using a flow cytometry procedure: 100 ml of basin water collected during shutdown was centrifuged at 17 000 × *g* for 30 min, after which the supernatant was discarded and the (invisible) cell pellet re-suspended in 1 ml of bottled Evian^®^ water filtered over a 0.2 μm Acrodisc syringe filter (Pall corporation). The solution was then stained with SYBR^®^ green (Life Technologies, Carlsbad, CA, United States), incubated for 20 min at 37°C and run on an Accuri^TM^ C6 flow cytometer (BD Biosciences, San Jose, CA, United States) in four replicates of 200 μl. Cells were manually gated on green fluorescence (FL1) vs. size (FSC) plots based on a negative control (0.2 μm filtered Evian^®^ water).

## Results

In order to study the dynamics of the bacterial community in the basin water, two sampling campaigns (separated by a 1-year interval) spanning multiple cycle and shutdown periods were performed using a 16S rRNA amplicon sequencing approach. The physico-chemical parameters of the water were monitored across both campaigns, whereas the cell number was assessed only during the second campaign. Finally, an experiment was designed to assess the effect of temperature on the basin water cell number.

### Physical Parameters

**FIGURE 1:**
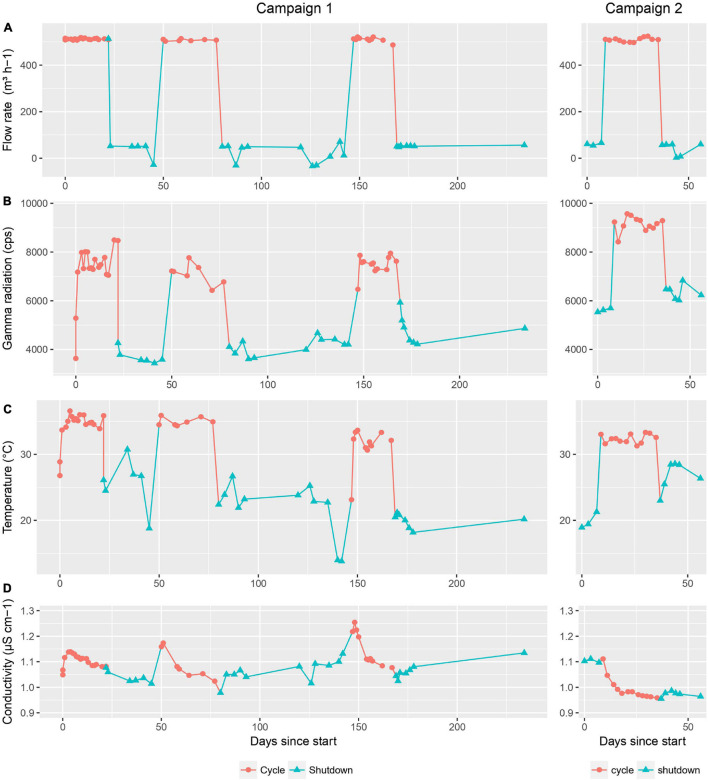
Physical parameters monitored during the first and second sampling campaign. **(A)** flow rate in the basin cooling circuit (m^3^ h^− 1^), **(B)** gamma radiation in the basin water (cps), **(C)** temperature in the basin water (°C). and **(D)** conductivity of the basin water (μS cm^− 1^). Cycle and shutdown periods are indicated by red circles and blue triangles, respectively.

During this study, four relevant physical parameters were monitored in order to potentially correlate those with the microbial community. The first parameter was the flow rate of the basin water cooling circuit (Figure 1A). During shutdown periods, the standard flow rate was 50 m^3^ h^–1^ while during operation it increased to 500 m^3^ h^–1^ to cool down the basin water. A second parameter was the global gamma radioactivity in the basin water (Figure 1B). As expected, the activity during operation significantly increased (up to a maximum of 9,500 cps) in comparison to shutdown periods (with a minimum of 3,500 cps) because of the nuclear fission reactions taking place in the reactor core. When the reactor is running, the radiation is deposited in the pool at a calculated dose rate of 755 Gy h^–1^, 98% of which is deposited in a zone within 5 cm around the core and 100 cm above and below the central plane. During shutdown periods, the dose rate lowers to 1 Gy h^–1^.

The third parameter was the temperature of the basin water (Figure 1C), which followed the same trend as observed for the other parameters: a significant increase during cycles (up to a maximum of 35°C), followed by a decrease to previous levels during shutdowns (with a minimum of 15°C). Lastly, the conductivity of the basin water also showed some variation across the timeline, although to a much smaller extent than the other parameters, with values ranging from 0.96 to 1.25 μS cm^–1^ (Figure 1D). As a side note, the flow rate in the purification circuit where deionization happens through mixed bed ion exchange resins remained constant throughout cycles and shutdown periods with a value of 30 m^3^ h^–1^.

### Chemical Analysis

The results of the chemical analysis performed on a selection of cycle and shutdown samples from campaign 1 can be found in Table 1. The concentrations of acetate and nitrate were extremely low, while the values for nitrite, phosphate, formate, TIC, and TOC were below the detection limit of the measuring instruments (10 μg l^–1^ for ion chromatography and 500 – 700 μg l^–1^ for TIC-TOC analysis, respectively). Furthermore, no significant difference between cycle and shutdown values could be detected. Other elements such as Zn, Pb, Co, Ni, Be, Fe, Mg, Cu, Al, Ba, Li, and F were also measured, but values were all below 20 μg l^–1^. pH was maintained at an average value of 6 ± 0.4. Finally, the total gamma activity across campaign 1 and 2 amounted to 1.9 × 10^3^ Bq l^–1^ on average during cycles and <80 Bq l^–1^ during shutdowns. 95% of the activity during cycles originates from Na-24, a radioisotope with a 15-h half-life. The remainder comes from other radioisotopes such as Cr-51, Sb-124, Re-186, Np-239, W-188, and Co-60.

**TABLE 1:** Chemical analysis performed on ecologically relevant compounds, during both cycle and shutdown periods.

**Component**	**Cycle (μg l^–1^)**	***n***	**Shutdown (μg l^–1^)**	***n***
Acetate	58 ± 16	5	67 ± 33	6
Nitrate	35 ± 3	5	44 ± 22	6
Nitrite	<10	5	<10	6
Phosphate	<10	5	<10	6
Formate	<10	5	<10	6
TIC	<500	3	<500	3
TOC	<700	3	<700	3

*Values represent mean ± standard deviation.*

### Cell Counting

**FIGURE 2:**
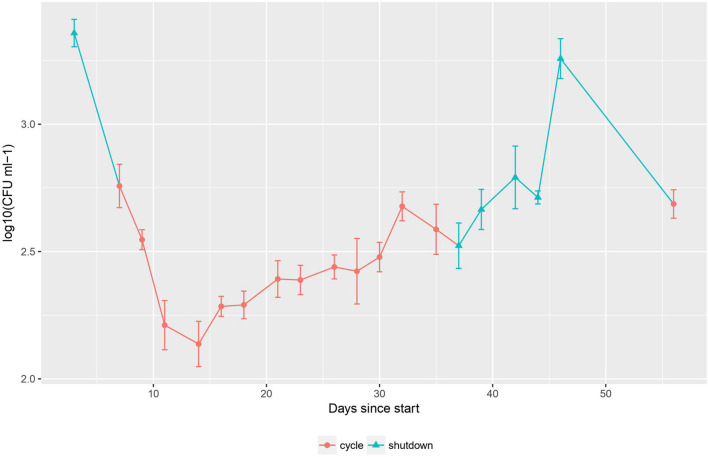
Cell counting during the second sampling campaign. Error bars represent standard deviation, *n* = 3. Cycle and shutdown periods are indicated by red circles and blue triangles, respectively.

In order to explore the effect of the changing physical conditions on the cell number of the bacterial population, heterotrophic plate count was used during the second sampling campaign (Figure 2). The data for this method follow a clear pattern: a steep decline in cell number of 1.2 log_10_ CFU ml^–1^ at the start of the cycle, followed by a stabilization and slow increase of the population by 0.5 log_10_ CFU ml^–1^ during the cycle and finally a recovery to previous cell density levels (up to 3.3 log_10_ CFU ml^–1^) after the cycle.

The observed pattern of cell number can be correlated with the change in physico-chemical parameters described previously. During a cycle, the flow rate of the cooling circuit for the basin water goes up from 50 to 500 m^3^ h^–1^. After passing through this circuit, the water is transferred back into the basin *via* a conduit following the reactor wall. At this point, the water is exposed to high levels of radiation originating from the nuclear fission taking place in the reactor core (gamma heating of 0.83 W g^–1^ = 830 Gy s^–1^ for a representative cycle of operation). Due to the high flow rate of the cooling circuit during a cycle, the water is frequently exposed to this radiation, with a turnover time of ca. 104 min.

### Bacterial Community Structure

The summary of the data analysis for the basin water samples given in Table 2 shows that the average number of OTUs for both campaigns did not significantly differ across cycles and shutdowns. Rarefaction curves for all samples are shown in Supplementary Figure 3.

**TABLE 2:** Summary of the data analysis performed for the first and second sampling campaigns.

	**Campaign 1**	**Campaign 2**
Sampling dates	06/09/2016 – 28/04/2017	16/04/2018 – 11/06/2018
Number of samples	61	21
Total number of reads	6,127,782	4,20,191
Average number of reads per sample	1,00,455	20,009
Minimum	55,044	11,683
Maximum	1,50,145	28,867
Standard deviation	18,990	3,611
Total number of OTUs	4,017	864
Average number of OTUs per sample	225	172

**FIGURE 3:**
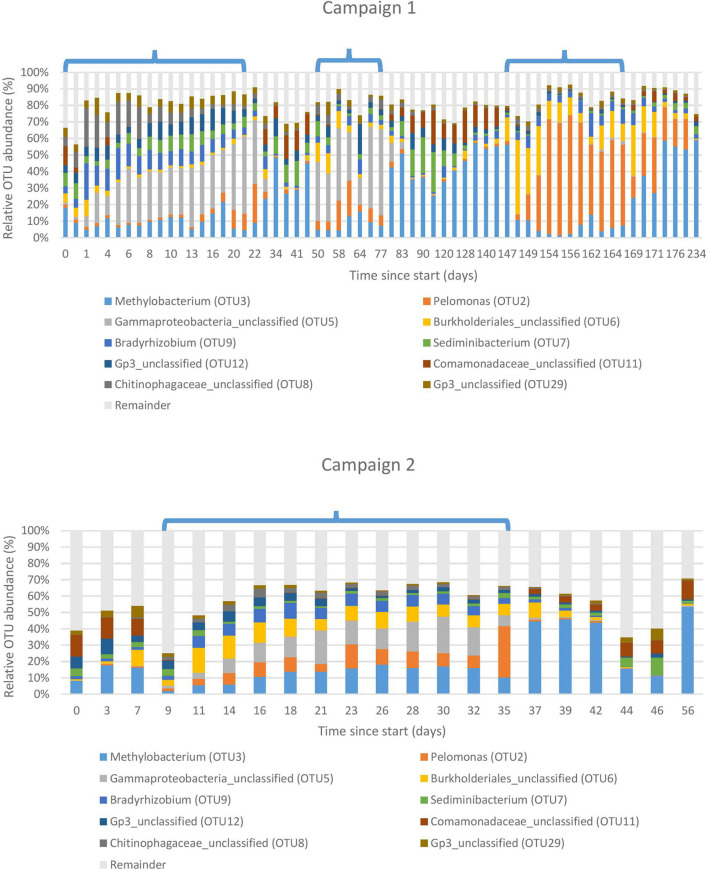
Bacterial community structure of the basin water environment. Ten most abundant Operational Taxonomic Units (OTUs) displayed in relative abundances. Each OTU is taxonomically assigned at the genus level. Remainder: All remaining OTUs not included in the 10 most abundant ones. Cycles are indicated by brackets, interspersed with shutdown periods. The horizontal axis indicates the number of days since the sampling start. Top: sampling campaign 1 (start-end: 06/09/2016-28/04/2017), bottom: sampling campaign 2 (start-end: 16/04/2018-11/06/2018).

Figure 3 shows the 10 most abundant OTUs in the basin water, representing 90 and 76% of the total reads for campaign 1 and 2, respectively. A clear shift in the community profile across the different cycle and shutdown periods is apparent. During the first sampling campaign, the most abundant OTU in the first two cycles was assigned to an unclassified *Gammaproteobacterium* (OTU5), accounting for 28% of total amplicon reads. For the third cycle, an OTU assigned to *Pelomonas* (OTU2) became predominant at 43%, although it could already be observed in lower proportions, at 5%, during the previous cycles. During the shutdown periods in between the cycles, the community profile shifted drastically and became dominated by an OTU assigned to *Methylobacterium* (OTU3) at 41%, while the previously mentioned OTUs (OTU5 and OTU2) were detected in much lower proportions (3 and 7%, respectively). This change can probably be assigned to the shift in physico-chemical parameters that occurs with the transition from a cycle to a shutdown period.

Although the second sampling campaign took place 1 year after the first one, the same pattern could be observed, indicating that the system is rather stable. Nevertheless, a difference can be noted for the unclassified *Gammaproteobacterium* and *Pelomonas* observed in approximately equal proportions (13 and 10%, respectively), whereas the community was either dominated by one or the other during the first sampling campaign.

### Temperature Effect on Cell Number

**FIGURE 4:**
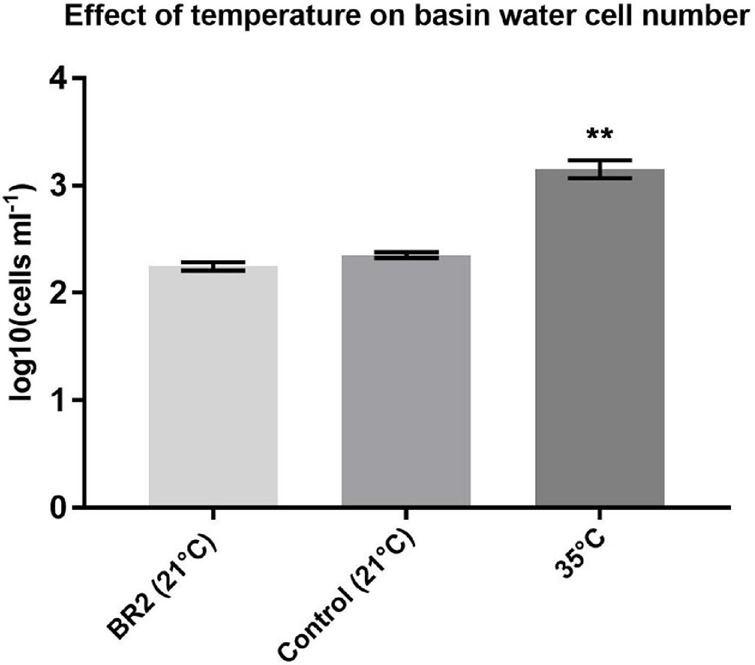
Effect of 24-h temperature increase on basin water cell number. Error bars represent standard error of the mean. Groups were compared using a non-parametric Kruskal–Wallis test. *P*-value = 0.0024, *n* = 4, **p < 0.01.

As can be observed in Figure 4, the cell number significantly increased when exposed to a higher temperature, indicating that temperature has an opposite effect to radiation. Since the overall cell number decreases during a cycle, this shows that the effect of radiation exposure largely outperforms the effect of temperature.

### Alpha and Beta Diversity Analyses

The alpha diversity showed a peak in diversity at the start of each cycle, followed by a subsequent downward trend (Supplementary Figure 4). This was true for both sampling campaigns, which further confirmed the robustness of the system under study.

**FIGURE 5:**
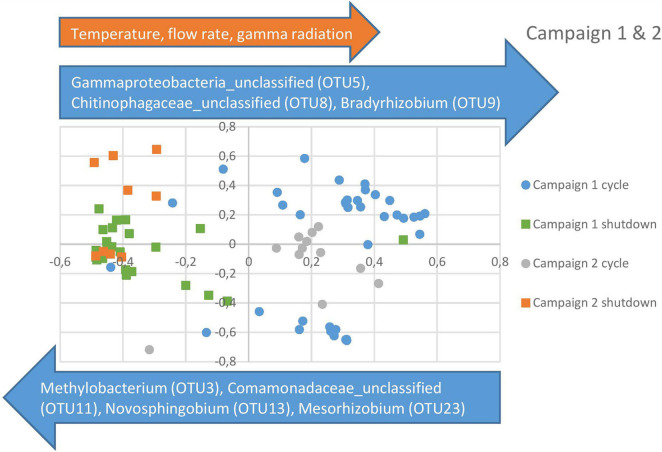
Non-metric Multidimensional Scaling (NMDS) for campaign 1 and 2 combined. The blue and orange arrows, respectively represent the OTUs and physico-chemical parameters showing a significant correlation (*r* > 0.6, *p* < 0.05) with the represented NMDS axes.

For the beta diversity analysis, an NMDS was performed on all the samples using the mothur program (Schloss et al., 2009). As can be observed in Figure 5, all shutdown samples from the first campaign clustered together, indicating that the community returns to an equilibrium after each cycle. Cycles 1 and 2 also clustered together in the top right quadrant, whereas cycle 3 was clustered separately in the bottom right quadrant, indicating a shift in the community at this point in time. This was also confirmed in Figure 3. Furthermore, the data points for the second campaign can be observed to blend in with the data points from campaign 1. Taking into consideration that the sampling campaigns were approximately 1 year apart, this further corroborates the fact that the system is very stable. Indeed, the shutdown data points for both campaigns were clustered together in the two left quadrants, whereas the cycle data points were clustered in the two right quadrants. Furthermore, the cycle from campaign 2 intercalates in between the cycles from the previous campaign, which supports a model where the bacterial community oscillates between different states.

In order to evaluate which parameters were responsible for the community shifts, a Spearman rank correlation analysis was performed between the data points on the NMDS axes and the observed OTUs and physico-chemical parameters. The OTUs with the largest impact on the separation between cycle and shutdown samples are represented as blue arrows in Figure 5. Regarding the physico-chemical parameters, it was observed that gamma radiation, flow rate, and temperature showed a significant correlation with the horizontal axis, whereas conductivity was marginally significant. Acetate and nitrate concentrations did not show a significant correlation. This suggests that radiation exposure, flow rate and temperature could be important factors in the community shift observed across cycles and shutdown periods. Correlation coefficients and *p*-values for the described OTUs and physico-chemical parameters can be found in Table 3.

**TABLE 3:** Summary of Spearman rank correlation analysis performed on Operational Taxonomic Units (OTUs) and physico-chemical parameters represented on the Non-metric Multidimensional Scaling (NMDS) plot.

**OTU**	**Average abundance**	***r***	***p*-value**	**Parameter**	***r***	***p*-value**
OTU5	11.39%	0.77	0	Temperature	0.71	0
OTU8	3.40%	0.70	0	Flow rate	0.63	0
OTU9	5.53%	0.62	0	Gamma radiation	0.62	0
OTU3	21.20%	–0.80	0	Conductivity	0.22	0.05
OTU11	3.76%	–0.84	0	Acetate	0.50	0.16
OTU13	2.06%	–0.69	0	Nitrate	0.32	0.37
OTU23	1.04%	–0.81	0			

## Discussion

The aim of the present study was to explore the bacterial community dynamics in the basin surrounding the core vessel of a nuclear research reactor. A first relevant observation was that the communities in the basin water underwent an apparent shift across cycle and shutdown periods. This was clearly observed in the community profiles and was further confirmed by the NMDS analysis, in which shutdown and cycle samples clustered separately. This shift correlated with a change in physico-chemical parameters such as temperature, flow rate and gamma radiation, whereas conductivity, nitrate, and acetate showed no significant correlation, indicating that the latter might have a lesser or no influence on the community composition. Other parameters such as pH and other ions were not included as these were either maintained at stable levels throughout the entire study or below the detection limit of the measuring instruments. The relevant parameters (individually or in combination) probably had an impact on the community shift from a population dominated by a member of the Gammaproteobacteria and *Pelomonas* during cycles to one dominated by *Methylobacterium* during shutdown periods. This pattern was observed for both sampling campaigns, indicating that this periodicity is stable. However, additional research is needed to elucidate the role of each individual parameter on the community, since all three of them follow the same pattern (high values during cycles and low values during shutdowns).

As such, temperature and radiation are believed to have the largest stress impact on the community. The observed shift during a transition from a shutdown period to a cycle could also be facilitated by the effect of stress on the community. Indeed, it has been previously described that the level of invasion of new species in communities with identical evenness depends strictly on the presence of stress (De Roy et al., 2013), here in the form of radiation exposure. In the absence of stress, communities tend to be more resistant to invasion.

The most abundant OTU during the third cycle of the first sampling campaign was *Pelomonas*, as opposed to the previous cycles that were mostly dominated by an unclassified Gammaproteobacterium. However, the cycle monitored during the second sampling campaign showed an approximately equal prevalence of both the unclassified Gammaproteobacterium and *Pelomonas*. This is reflected in the NMDS analysis, where the data points of the campaign 2 cycle intercalate in the middle of the other cycle data points with little overlap. This suggests that the community during cycles can fluctuate between different states, either equally dominated by the two main OTUs or only dominated by one of them. Taking into consideration that the physico-chemical parameters across different cycles remain constant, this phenomenon can probably be attributed to stochastic effects, which might be investigated in future research. Indeed, it is known that the mechanisms governing community assembly can be both deterministic and stochastic in nature (Sloan et al., 2006; Nemergut et al., 2013; Evans et al., 2017; Zhou and Ning, 2017). As such, next to deterministic factors like species traits, interspecies interactions and environmental conditions, stochastic processes in the form of random birth and death events, colonization and extinction are also known to play a role in this regard. Furthermore, it has been shown that a single source community can, under identical environmental conditions, generate drastically different communities with little overlap in composition or even global function (Zhou et al., 2013).

The chemical analysis of the water samples showed that all of the measured components (nitrite, phosphate, formate, TIC, and TOC as well as other chemical elements) except for acetate and nitrate were below the detection limit of the measuring instruments. This is due to the fact that the water is constantly being deionized through ion exchangers, resulting in a very low conductivity of approximately 1 μS cm^–1^ and an extremely oligotrophic environment. However, even in ultrapure waters, it is impossible to eliminate all impurities. In environments such as the one monitored in our study, these impurities mainly originate from metallic pipings in the cooling and purification circuits, dust that falls into the basin and the air in contact with the pool surface. Typical dominant impurities include Al_3_^+^, Fe_2_^+^, NO_3_^−^, SO_42_^−^ and Cl^−^ (International Atomic Energy Agency, 2011). These can in turn be utilized by bacterial communities. Furthermore, the basin of the BR2 research reactor is regularly emptied during shutdown periods for maintenance purposes, allowing technicians to access areas that would normally be submerged. This could periodically introduce organic material in the system, which could be used as an energy source by heterotrophic bacteria.

Another source of organic material could be the ion exchange resins used in the purification circuit. These are mixed bed resins composed of polystyrene beads that absorb cations and anions on their surface. Due to radiation, these resins slowly degrade over time (approximately 5 years), hereby releasing small quantities of organic material into the water, which can then be used by bacteria. In addition, they could also be a substrate for bacterial growth, as has been demonstrated by one particular research group (Kéki et al., 2019).

The basin is an oxic environment, since it is an open system with high water circulation (50 – 500 m^3^ h^–1^) which allows for a homogeneous oxygen distribution. This is also evidenced by the fact that most identified genera based on the 16S rRNA amplicon sequencing data are strictly or facultatively aerobic. In addition, the presence of ionizing radiation causes water molecules to dissociate into several chemical species such as H_2_, H_2_O_2_, and some radicals (OH•, H•, and HO_2_•), a phenomenon known as water radiolysis (Dzaugis et al., 2015). These characteristics combined with the low nutrient availability result in some unique conditions for which different bacteria have adopted different strategies. For example, it has been previously demonstrated that bacteria in SNFPs can oxidize H_2_ from water radiolysis as an energy source, using O_2_ as electron acceptor and CO_2_ as carbon source (Galès et al., 2004). As such, the *Pelomonas* and *Bradyrhizobium* genera as well as the Comamonadaceae family detected in our system have been associated with chemoautotrophic growth on H2 (Willems et al., 1991; Gomila et al., 2007; Franck et al., 2008). On the other hand, *Methylobacterium* is a facultative methylotroph known to be able to grow on C2 compounds such as acetate as a sole carbon source (Smejkalova et al., 2010; Green and Ardley, 2018). *Bradyrhizobium* and *Mesorhizobium* on the other hand are capable of N^2^ fixation (Black et al., 2012). It must also be noted that bacteria in oligotrophic environments can rely on mixotrophy as a survival strategy, since this provides them with a greater nutritional flexibility (Mittelman and Jones, 2018). Furthermore, the above-described taxa were also detected in different SNFPs and other cooling basins using 16S rRNA amplicon sequencing (Bagwell et al., 2018; MeGraw et al., 2018; Petit, 2018; Petit et al., 2020). This can probably be attributed to the fact that the physico-chemical parameters in SNFPs and the basin under study are quite similar. Indeed, the water in SNFPs is also very oligotrophic due to constant deionization *via* anion and cation exchangers and is exposed to residual levels of radiation originating from the spent fuel elements stored underwater.

In addition, we propose that the established community may be able to recycle dead cell material in order to survive long periods of nutrient deprivation. Indeed, it has been previously demonstrated that bacteria can survive for extended periods of time under conditions of starvation, and that this phenomenon is inversely proportional to cell density (Phaiboun et al., 2015). Moreover, bacteria in their natural environments are believed to exist in conditions resembling those of long-term stationary phase cultures, where stress-response genes and alternative metabolic pathways are essential for survival (Finkel, 2006). It has also been shown that cell death and recycling were essential processes for cell survival during starvation, where survivors were able to utilize nutrients from dead cells parsimoniously as a strategy for long-term persistence (Takano et al., 2017). In our system, the cell number rapidly decreases with a magnitude of more than one log during the transition from a shutdown period to a cycle (Figure 2). This cell number decrease could be assigned to the water being exposed to high levels of radiation originating from the reactor core. We therefore propose that cells that have been killed off by radiation are rapidly being recycled by the surviving population for nutrient retrieval. This phenomenon could also partially explain the peak in diversity observed at the start of each cycle (Supplementary Figure 4). Indeed, the temporary availability in nutrients could allow other species to emerge, resulting in a brief increase in both richness and evenness before the community reverts toward an equilibrium state characterized by a lower diversity. The stressing effect of radiation on the community could also account for the downward trend in diversity observed for both sampling campaigns. This could potentially be investigated in further research.

In this study, we mainly focused on the characterization of the bacterial community, but it could also be of interest to investigate the presence of eukaryotes such as micro-algae and fungi through 18S and ITS amplicon sequencing. Our study could also be complemented with additional research investigating the radiation resistance of individual strains isolated from this environment or of the entire community as a whole. In addition, it would be of interest to perform a deeper analysis on the community using NGS techniques such as shotgun metagenomics and metaproteomics. This would allow for the development of a deeper insight into the specific genes and proteins responsible for the behavior of the community in this unique environment.

## Data Availability Statement

The datasets presented in this study can be found online in the BioProject repository (https://www.ncbi.nlm.nih.gov/bioproject/) under accession number PRJNA725077.

## Author Contributions

VVE performed the sampling, laboratory work and data analysis, and wrote the manuscript. RP provided advice on the research design and flow cytometry. MM contributed to the 16SrRNA sequencing data processing. PM provided advice on the research design and data interpretation. All authors critically reviewed the manuscript.

## Conflict of Interest

The authors declare that the research was conducted in the absence of any commercial or financial relationships that could be construed as a potential conflict of interest.

## Publisher’s Note

All claims expressed in this article are solely those of the authors and do not necessarily represent those of their affiliated organizations, or those of the publisher, the editors and the reviewers. Any product that may be evaluated in this article, or claim that may be made by its manufacturer, is not guaranteed or endorsed by the publisher.
