# Supplementary material for: Cyclical Patterns Affect Microbial Dynamics in the Water Basin of a Nuclear Research Reactor
Source: Front Microbiol. 2021 Oct 15;12:744115. doi: 10.3389/fmicb.2021.744115 (PMC8555696; doi:10.3389/fmicb.2021.744115)
Supplement: Supplementary file 1 [file Data_Sheet_1.docx]

Supplementary Material

**
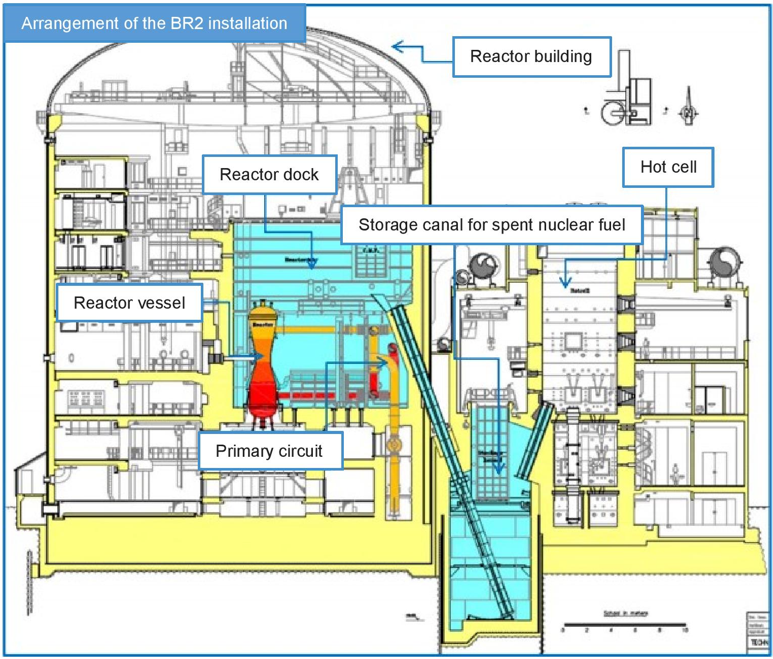
**

**Supplementary figure S1.** **Schematic overview of the BR2 facility.** Watery environments are represented in blue, orange and red. Reactor vessel color-coded in shades of orange and red in function of the temperature in the primary circuit water.

**
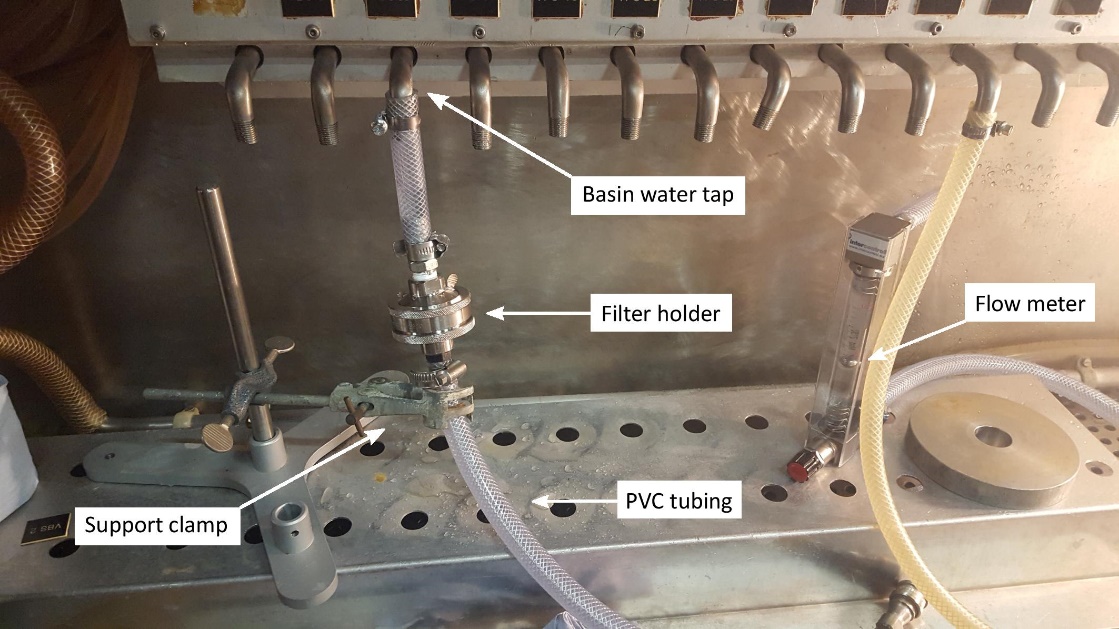
**

**Supplementary figure S2.** **Filtration system implemented in a sampling glove box in the BR2 reactor.** Individual components are indicated by arrows.


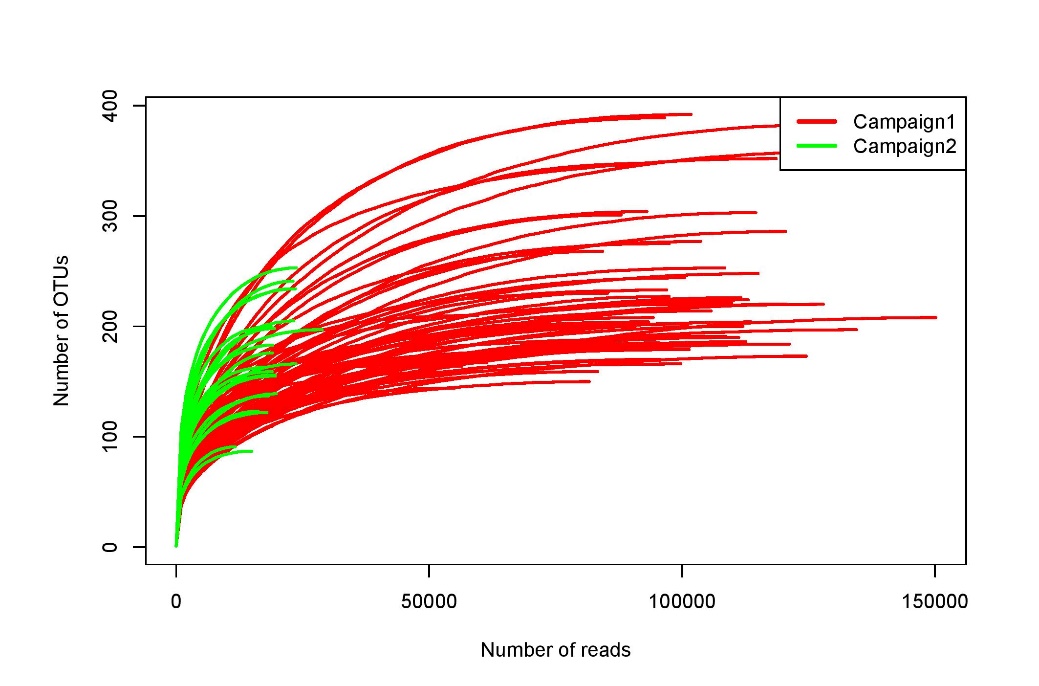


**Supplementary figure S3.** **Rarefaction curves for all samples of campaign 1 and 2.**

**
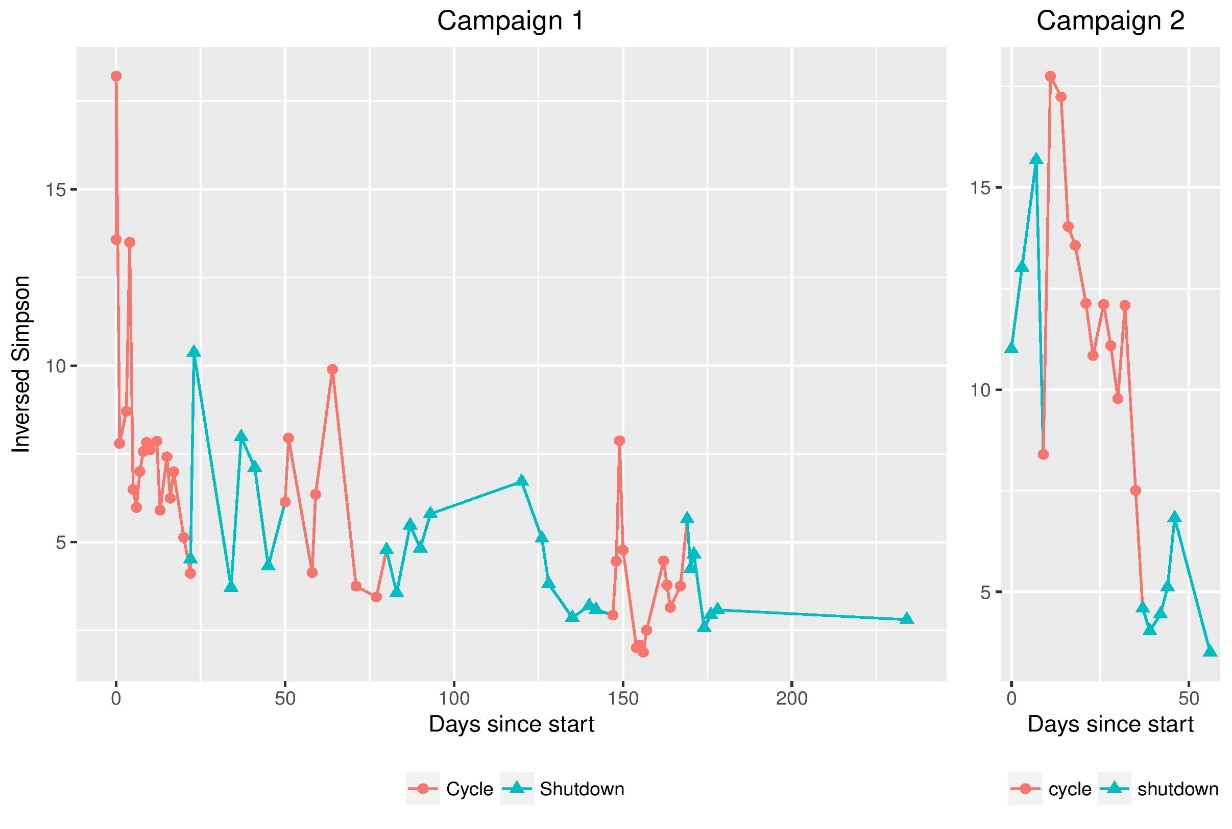
**

**Supplementary figure S4. Alpha diversity plots (inversed Simpson diversity index).** Left: Campaign 1, right: Campaign 2. The horizontal axis represents the number of days since the sampling start. Cycle and shutdown periods are indicated by red circles and blue triangles, respectively.

**Supplementary table S1.** **Sample metadata for campaign 1 and 2.**

| Sample ID | Campaign | Sampling date | Days since start | Operational status |
| --- | --- | --- | --- | --- |
| BW7_1 | 1 | 2016-09-06 | 0 | Cycle |
| BW8_1 | 1 | 2016-09-06 | 0 | Cycle |
| BW9_1 | 1 | 2016-09-07 | 1 | Cycle |
| BW10_1 | 1 | 2016-09-09 | 3 | Cycle |
| BW11_1 | 1 | 2016-09-10 | 4 | Cycle |
| BW12_1 | 1 | 2016-09-11 | 5 | Cycle |
| BW13_1 | 1 | 2016-09-12 | 6 | Cycle |
| BW14_1 | 1 | 2016-09-13 | 7 | Cycle |
| BW15_1 | 1 | 2016-09-14 | 8 | Cycle |
| BW16_1 | 1 | 2016-09-15 | 9 | Cycle |
| BW17_1 | 1 | 2016-09-16 | 10 | Cycle |
| BW18_1 | 1 | 2016-09-18 | 12 | Cycle |
| BW19_1 | 1 | 2016-09-19 | 13 | Cycle |
| BW21_1 | 1 | 2016-09-21 | 15 | Cycle |
| BW22_1 | 1 | 2016-09-22 | 16 | Cycle |
| BW23_1 | 1 | 2016-09-23 | 17 | Cycle |
| BW24_1 | 1 | 2016-09-26 | 20 | Cycle |
| BW25_1 | 1 | 2016-09-28 | 22 | Cycle |
| BW26_1 | 1 | 2016-09-28 | 22 | Shutdown |
| BW27_1 | 1 | 2016-09-29 | 23 | Shutdown |
| BW29_1 | 1 | 2016-10-10 | 34 | Shutdown |
| BW30_1 | 1 | 2016-10-13 | 37 | Shutdown |
| BW31_1 | 1 | 2016-10-17 | 41 | Shutdown |
| BW32_1 | 1 | 2016-10-21 | 45 | Shutdown |
| BW33_1 | 1 | 2016-10-26 | 50 | Cycle |
| BW34_1 | 1 | 2016-10-27 | 51 | Cycle |
| BW35_1 | 1 | 2016-11-03 | 58 | Cycle |
| BW36_1 | 1 | 2016-11-04 | 59 | Cycle |
| BW37_1 | 1 | 2016-11-09 | 64 | Cycle |
| BW38_1 | 1 | 2016-11-16 | 71 | Cycle |
| BW39_1 | 1 | 2016-11-22 | 77 | Cycle |
| BW40_1 | 1 | 2016-11-25 | 80 | Shutdown |
| BW41_1 | 1 | 2016-11-28 | 83 | Shutdown |
| BW42_1 | 1 | 2016-12-02 | 87 | Shutdown |
| BW43_1 | 1 | 2016-12-05 | 90 | Shutdown |
| BW44_1 | 1 | 2016-12-08 | 93 | Shutdown |
| BW45_1 | 1 | 2017-01-04 | 120 | Shutdown |
| BW46_1 | 1 | 2017-01-10 | 126 | Shutdown |
| BW47_1 | 1 | 2017-01-12 | 128 | Shutdown |
| BW49_1 | 1 | 2017-01-19 | 135 | Shutdown |
| BW50_1 | 1 | 2017-01-24 | 140 | Shutdown |
| BW51_1 | 1 | 2017-01-26 | 142 | Shutdown |
| BW52_1 | 1 | 2017-01-31 | 147 | Cycle |
| BW53_1 | 1 | 2017-02-01 | 148 | Cycle |
| BW54_1 | 1 | 2017-02-02 | 149 | Cycle |
| BW55_1 | 1 | 2017-02-03 | 150 | Cycle |
| BW56_1 | 1 | 2017-02-07 | 154 | Cycle |
| BW57_1 | 1 | 2017-02-08 | 155 | Cycle |
| BW58_1 | 1 | 2017-02-09 | 156 | Cycle |
| BW59_1 | 1 | 2017-02-10 | 157 | Cycle |
| BW60_1 | 1 | 2017-02-15 | 162 | Cycle |
| BW61_1 | 1 | 2017-02-16 | 163 | Cycle |
| BW62_1 | 1 | 2017-02-17 | 164 | Cycle |
| BW63_1 | 1 | 2017-02-20 | 167 | Cycle |
| BW64_1 | 1 | 2017-02-22 | 169 | Shutdown |
| BW65_1 | 1 | 2017-02-23 | 170 | Shutdown |
| BW66_1 | 1 | 2017-02-24 | 171 | Shutdown |
| BW67_1 | 1 | 2017-02-27 | 174 | Shutdown |
| BW68_1 | 1 | 2017-03-01 | 176 | Shutdown |
| BW69_1 | 1 | 2017-03-03 | 178 | Shutdown |
| BW71_1 | 1 | 2017-04-28 | 234 | Shutdown |
| BW0_2 | 2 | 2018-04-16 | 0 | Shutdown |
| BW1_2 | 2 | 2018-04-19 | 3 | Shutdown |
| BW2_2 | 2 | 2018-04-23 | 7 | Cycle |
| BW3_2 | 2 | 2018-04-25 | 9 | Cycle |
| BW4_2 | 2 | 2018-04-27 | 11 | Cycle |
| BW5_2 | 2 | 2018-04-30 | 14 | Cycle |
| BW6_2 | 2 | 2018-05-02 | 16 | Cycle |
| BW7_2 | 2 | 2018-05-04 | 18 | Cycle |
| BW8_2 | 2 | 2018-05-07 | 21 | Cycle |
| BW9_2 | 2 | 2018-05-09 | 23 | Cycle |
| BW10_2 | 2 | 2018-05-12 | 26 | Cycle |
| BW11_2 | 2 | 2018-05-14 | 28 | Cycle |
| BW12_2 | 2 | 2018-05-16 | 30 | Cycle |
| BW13_2 | 2 | 2018-05-18 | 32 | Cycle |
| BW14_2 | 2 | 2018-05-21 | 35 | Cycle |
| BW15_2 | 2 | 2018-05-23 | 37 | Shutdown |
| BW16_2 | 2 | 2018-05-25 | 39 | Shutdown |
| BW17_2 | 2 | 2018-05-28 | 42 | Shutdown |
| BW18_2 | 2 | 2018-05-30 | 44 | Shutdown |
| BW19_2 | 2 | 2018-06-01 | 46 | Shutdown |
| BW20_2 | 2 | 2018-06-11 | 56 | Shutdown |
